# Aqueous Solubility and Degradation Kinetics of the Phytochemical Anticancer Thymoquinone; Probing the Effects of Solvents, pH and Light

**DOI:** 10.3390/molecules19055925

**Published:** 2014-05-08

**Authors:** Jumah Masoud M. Salmani, Sajid Asghar, Huixia Lv, Jianping Zhou

**Affiliations:** 1 Department of Pharmaceutics, School of Pharmacy, China Pharmaceutical University, 24 Tongjiaxiang, Nanjing 210009, China; E-Mail: sajuhappa@gmail.com; 2 College of Pharmacy, Al-Mustansiriya University, Al-Qadisiya District, Baghdad 10015, Iraq; 3 College of Pharmacy, Government College University Faisalabad, Faisalabad 38000, Pakistan

**Keywords:** thymoquinone, phytochemical, stability, degradation kinetics, solubility

## Abstract

Thymoquinone (TQ) is a potent anticancer phytochemical with confirmed *in vitro* efficacy. Its clinical use has not yet established, and very few reports have documented its formulation. There also are no reports about the aqueous solubility and stability of this valuable drug, despite their direct correlation with the *in vivo* efficacy. In the current research, we have established and validated a stability-indicating HPLC method for the detection of TQ and its degradation products under different conditions. We then investigated the solubility and stability profiles of TQ in aqueous solutions. The stability study was aimed to determine the effect of pH, solvent type and light on the degradation process of TQ, along with the investigation of the kinetics of this degradation. The solubility of TQ varied in different aqueous solvents, and might be compromised due to stability issues. However, these findings confirm that the aqueous solubility is not the major obstacle for the drug formulations mainly due to the considerable water solubility (>500 μg/mL) that may be enough to exert pharmacologic effects if administered via parenteral route. Stability study results showed a very low stability profile of TQ in all the aqueous solutions with rapid degradation that varied with solvent type. The study of the degradation kinetics showed a significant effect of pH on the degradation process. The process followed first order kinetics at more acidic and alkaline pH values, and second order kinetics at pH 5–7.4, regardless of the solvent type. The results also expressed that light has a greater impact on the stability of TQ as a shorter period of exposure led to severe degradation, independent of the solution pH and solvent type. Our results also addressed some discrepancies in previously published researches regarding the formulation and quantification of TQ with suggested solutions. Overall, the current study concludes that TQ is unstable in aqueous solutions, particularly at an alkaline pH, in addition to presenting severe light sensitivity. This data indicates the inappropriateness of aqueous solutions as pharmaceutical vehicles for TQ preparations. To the best of our knowledge, this is the first study describing TQ aqueous solubility and stability that may lead to the development of a stable and effective TQ formulation.

## 1. Introduction

Thymoquinone (TQ) is the main bioactive constituent of an oil extract of *Nigella sativa*. The last fifteen years have witnessed hundreds of research reports regarding its therapeutic biological effects as an anti-inflammatory, analgesic, anti-diabetic, antihistaminic and anticancer agent [1]. TQ exerts its biological functions by modulating the physiological and biochemical processes involved in reactive oxygen species (ROS) generation both in normal and tumor cells where it acts as antioxidant and pro-oxidant, respectively [1,2,3,4,5,6,7]. In the prognosis of cancer, the cells must have some features (hallmarks) in order to enable their transformation to malignant tumors [8]. TQ has been proved to affect nine out of the ten known cancer hallmarks, and drugs that affect or modulate even one of them should be considered as good candidates for clinical trials [9]. However, to date TQ has not been used in clinical trials, mainly due to formulation problems. The already reported formulations of TQ clearly share problems of low drug loading and burst release [10,11,12,13,14,15,16], which seriously compromises its *in vivo* efficacy. Additionally the lack of information regarding the stability of the drug in those formulations or during the course of production and later through the shelf life, constitute a major problem in drug formulation. Thus, preforming these studies is crucial because it will assist in understanding the physicochemical properties of the drug and may provide the foundation for the development of a robust dosage form. Although, some physicochemical properties of TQ had been previously investigated [17,18,19], a literature survey revealed the lack of solubility and stability studies despite their major contribution to the formulation of an effective dosage form. In this report, we studied the solubility, stability and kinetics of degradation of TQ in different solutions along with the effect of pH and light. We expect this study to be helpful in future research in the field of TQ formulations that may accelerate its clinical applications.

## 2. Results and Discussion

### 2.1. Qualitative UV/VIS Spectrophotometric Analysis

**Figure 1:**
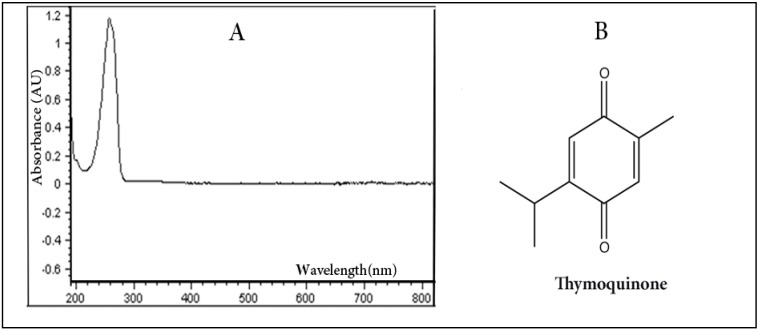
The UV-VIS spectrum of TQ (10 μg/mL) in ethanol (**A**) and the chemical structure of the drug (**B**).

The wavelengths of absorption peaks can be correlated with the types of bonds in a given molecule and are valuable in determining the functional groups present within a molecule. The UV-VIS spectra of thymoquinone is characterized by the presence of one prominent peak (λ_max_) at 254–257 nm as shown in Figure 1, regarded as a distinguishing peak for quinones from its analog hydroquinone where the latter shows a peak near 290 nm [20]. The spectra have been recorded using concentration of 10 μg/mL. By having such a prominent peak at this concentration, the use of this wavelength for the detection of TQ in HPLC method would make it highly sensitive even to very low concentrations of TQ. Although 294 nm was used in previous study for the detection of TQ [21], our UV-VIS results showed that TQ has no significant absorption at this wavelength, even with the same mobile phase combination.

### 2.2. Quantitative Analysis by HPLC

The method was found to be linear in the range of 0.5–10 μg/mL, with a mean R^2^ value of 0.9999 with the linear equation of y = 3.34 × 10^5^*x* + 2.69 × 10^4^.

**Figure 2:**
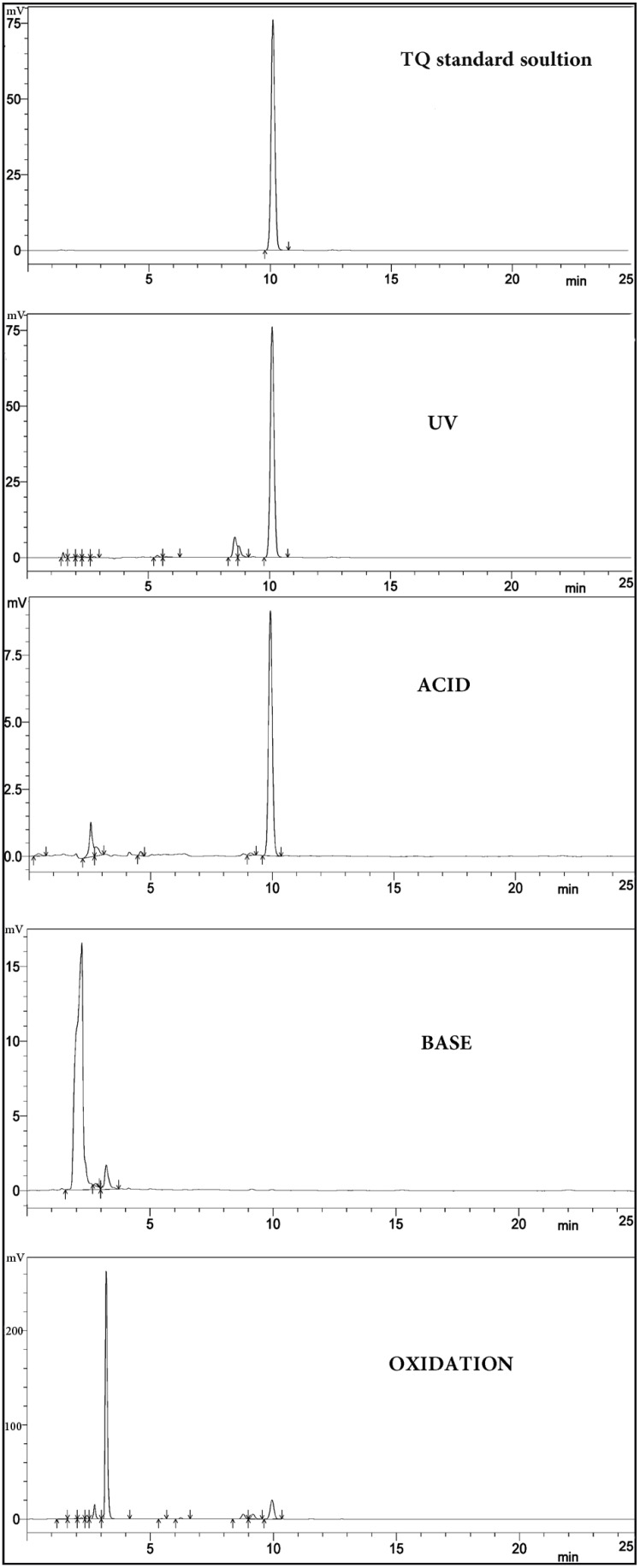
HPLC chromatograms of the forced degradation study of TQ in acid, base, UV, and oxidation stress conditions compared to the standard solution of TQ prepared in mobile phase.

Method Specificity is the ability to evaluate unequivocally the analyte in the presence of other components, such as impurities and degradants. The peak of TQ was retained at 10 min, quite far away from any interference from other impurities, so apparently, no interference was found at the retention time of TQ (Figure 2).

The precision of an analytical procedure is usually expressed as the standard deviation or coefficient of variation of a series of measurements (Relative Standard Deviation RSD %). The results of inter-day and intra-day variation illustrated in Table 1 show the process for the determination of TQ from bulk solutions was highly precise and repeatable. The inter-day and intra-day precision values for the proposed method were found to be <8% for the lowest concentration and <2% for the highest concentration tested. The Limit Of Detection (LOD) and Limit Of Quantitation (LOQ) calculated from the calibration curve were 0.08 and 0.246 µg/mL, respectively.

**Table 1:** HPLC method precision evaluation, the repeatability (intra-day) and intermediate precision (inter-day) represented by SD and RSD% (standard deviation and the relative standard deviation) for a series of measurements.

Conc. μg/mL	Intra-day			Inter-day		
	Mean	SD	RSD%	Mean	SD	RSD%
0.5	0.5116	0.0368	7.2007	0.4672	0.0365	7.8063
1	1.0392	0.0837	8.0586	0.9736	0.0464	4.7630
2	2.0381	0.0342	1.6775	1.9635	0.0633	3.2219
4	4.0056	0.0485	1.2096	3.9289	0.1201	3.0579
6	5.9810	0.0523	0.8740	5.9191	0.1263	2.1337
8	7.8856	0.0438	0.5557	7.7934	0.1990	2.5530
10	9.9475	0.1498	1.5058	9.7306	0.1849	1.9001

### 2.3. Forced Degradation Study

According to ICH regulations stress testing of a drug substance can help identify the likely degradation products, which can in turn help establish the degradation pathways and the intrinsic stability of the molecule and validate the stability indicating power of the analytical procedures used. The nature of the stress testing will depend on the individual drug substance and the type of drug product involved. In the current research, forced degradation studies were conducted in order to ensure our method’s fitness for the claim of suitability as a stability indicating method. Figure 2 shows the results of the forced degradation study compared to the standard TQ solution. Under acidic conditions, TQ suffered minimum degradation as indicated by the appearance of small peaks before the peak of TQ. Under basic conditions, TQ was rapidly degraded and its corresponding peak at 10 min disappeared totally from the chromatograms and instead the degradation peaks appeared much earlier. The retention time for the acid and base degradation products are different, indicating the different nature of each product formed from these processes. Due to their earlier retention time, we can state that they share the similar property of being more hydrophobic than TQ.

The UV and oxidative forced degradation studies thus revealed a number of degradation peaks, well resolved from that of TQ, all of which appear earlier to the drug in the HPLC chromatograms. These results confirm the suitability of the HPLC method as a stability-indicating method for the quantification of TQ and its degradation products.

### 2.4. The Solubility Study

Solubility is often signified as a characteristic property and as a guide to the applications of a substance. A review of the relevant literature shows a lack of specific data about TQ solubility in commonly used solvents, thus performing the solubility study in such solvents is essential for a successful formulation process. Table 2 summarizes the results of the solubility study. In 24 h, TQ shows a comparatively similar solubility ranging from 549–669 μg/mL in all aqueous solutions. Although, the solubility increased up to 72 h except under extreme acidic and basic solutions, it was not possible to specify a certain value for the solubility of TQ in these solvents, most probably due to chemical and/or physical instability of the drug in these solvents that may interfere with the soluble drug fraction. Further studies are therefore needed to explore the possible degradation pathways and the resultant products in each solvent in order to have a better understanding of the noted fluctuations in the solubility behavior.

**Table 2:** Summary of the solubility study results, the values represent the concentration of TQ ± SD in μg/mL, H (hours), P.B (phosphate buffer).

Time (H)	Water	0.1 N HCl	P.B pH 5	P.B pH 7.4	P.B pH 9
24 h	669.13 ± 5.4	653.64 ± 1.9	619 ± 7.6	566.43 ± 0.19	549.19 ± 0.46
48 h	698.16 ± 1.4	638.4 ± 2.93	720.1 ± 1.57	550.12 ± 1.87	727.57 ± 4.69
72 h	740.63 ± 5	478.48 ± 0.96	739.20 ± 12	610.88 ± 4.5	665.66 ± 17.5

However, the observed solubility profile in aqueous solutions leads us to conclude that TQ possesses good water solubility that is enough to maintain effective therapeutics levels *in vivo* in the case of parenteral use. The aim behind TQ formulations should therefore not focus on increasing the water solubility but rather on improving the stability of the soluble drug.

### 2.5. Aqueous Stability Study

**Figure 3:**
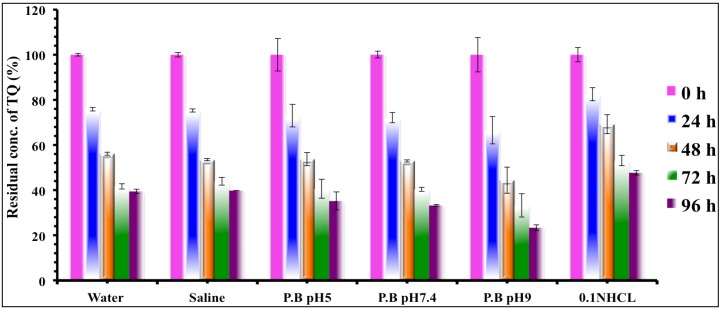
Residual TQ concentration in different solutions at variable time intervals of the stability study estimated by HPLC, Time in hours, (error bars represent the standard deviation).

**Figure 4:**
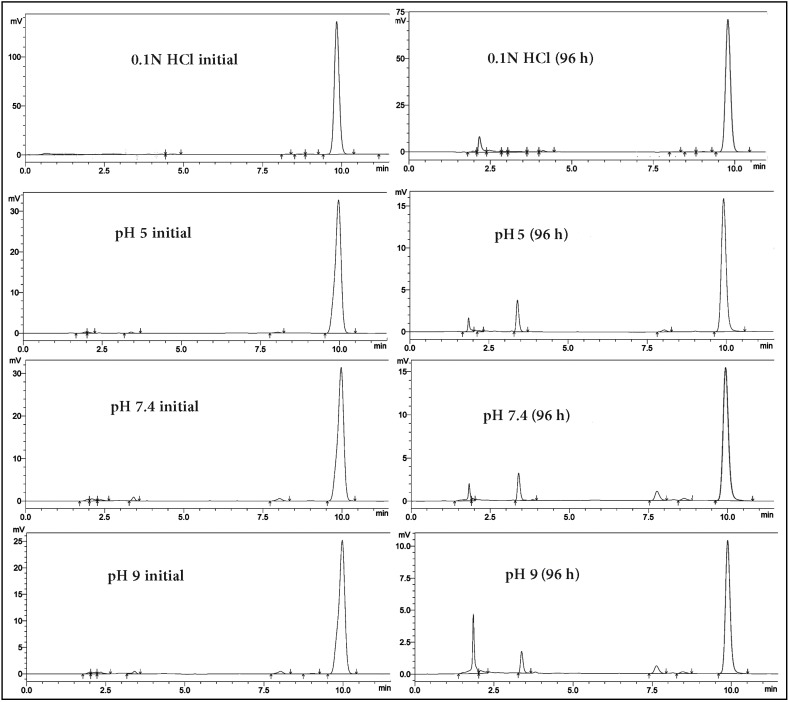
HPLC chromatograms of TQ before and after four days of the stability study in different solutions, showing the decrease in the peak intensity of the drug and the appearance of new degradation peaks.

In order to evaluate the stability of TQ in aqueous solutions with different pH values we used 0.1 N HCl solution and three phosphate buffer solutions (pH 5, 7.4 and 9) together with water and saline solution. High performance liquid chromatography (HPLC) was used to trace the drug concentrations throughout the study; the chromatograms of different solvents were compared at each time point. Results illustrated in Figure 3 show a continuous decrease in the concentration of the drug with time in all the tested solutions. Figure 4 shows the chromatograms of the tested solutions initially compared with those after 96 h. The Area Under the Curve (AUC) of the peak corresponding to the parent drug (TQ) was noticed to decline, accompanied by the appearance of new degradation peaks. These peaks appeared at 2–2.3, 3.4, 7.7, and 8 min, their intensities varied depending on the tested solution. The degradation rate at the acidic pH was very minor compared to other solutions, indicating that TQ at acidic pH suffered minimal degradation.

**Figure 5:**
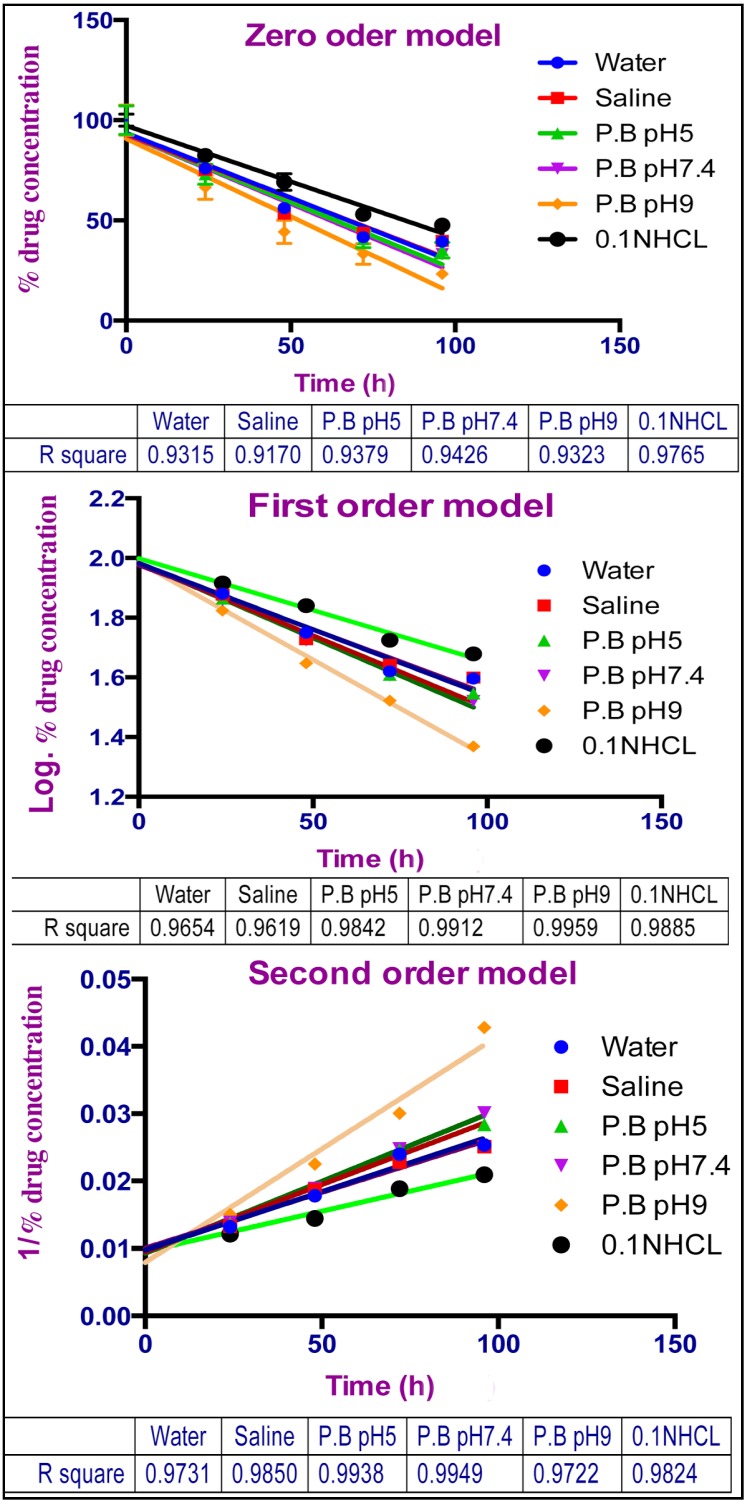
Degradation kinetics of TQ in aqueous media, zero order, First order, and second order kinetics models.

A similar degradation rate of TQ was noticed in both water and saline solutions, and the same effect but with a slightly higher value was found in phosphate buffer solutions (pH 5 and pH 7.4). The highest degradation rate was observed at alkaline pH. The degradation peaks were also noticed to increase both in number and intensity with increasing the pH value. In order to understand the kinetics of the degradation, the data was plotted for zero, first and second order kinetics as well as the R^2^ values were compared (Figure 5).

From the values of R^2^, we can recognize that TQ degradation at lower and higher pH (0.1 N HCl and pH 9) followed first order kinetics, which means the degradation mainly depends on the available TQ concentration. Solutions of pH 5, water, saline, and pH 7.4 were found to follow second order kinetics, allowing us to assume that the degradation process in these solutions not only depends on the concentration of TQ but also is affected by either the pH of the solution or the presence of phosphate salts, indicating the involvement of more than one factor in the degradation process.

From this data, we conclude that TQ is more stable at lower pH and its stability decreases with rising pH. This was in agreement with the forced degradation study performed by us and also reported earlier [21] where TQ suffered minimal degradation at acidic pH with complete degradation at highly alkaline pH.

Overall results indicate that TQ underwent severe degradation in aqueous media and the structure elucidation of the degradation products in each solvent may provide a better understanding to the mechanism of degradation and may offer possible prevention measures. Such research is beyond the scope of the current work and might be considered in the future. The current study provides enough evidence for the unsuitability of using aqueous media for the handling and quantification of TQ. Apparently, this had already led to discouraging results in TQ formulations, such as the discrepancies in using aqueous solutions as a release media for the estimation of TQ. The negligible drug release in the aqueous media, especially for the formulation with low drug loading [13], could be due to the degradation of TQ in aqueous media.

We also relate this phenomena to the unexplained decline in the released amount of TQ with time, reported elsewhere [14]. The observed rapid drug degradation also leads to questions about the accuracy of the results obtained through the use of aqueous media for the determination of TQ release from nanoparticles. If the release rate is more than the degradation rate, there will be a net drug concentration available for detection, but with time, the rate of release may decline and the net result may be in favor for the degradation. Due to loss of some drug (degraded part) the overall results throughout the experimental period may not represent the actual values of the drug released.

### 2.6. Effect of Light and Solvent Type on the Degradation of TQ

**Figure 6:**
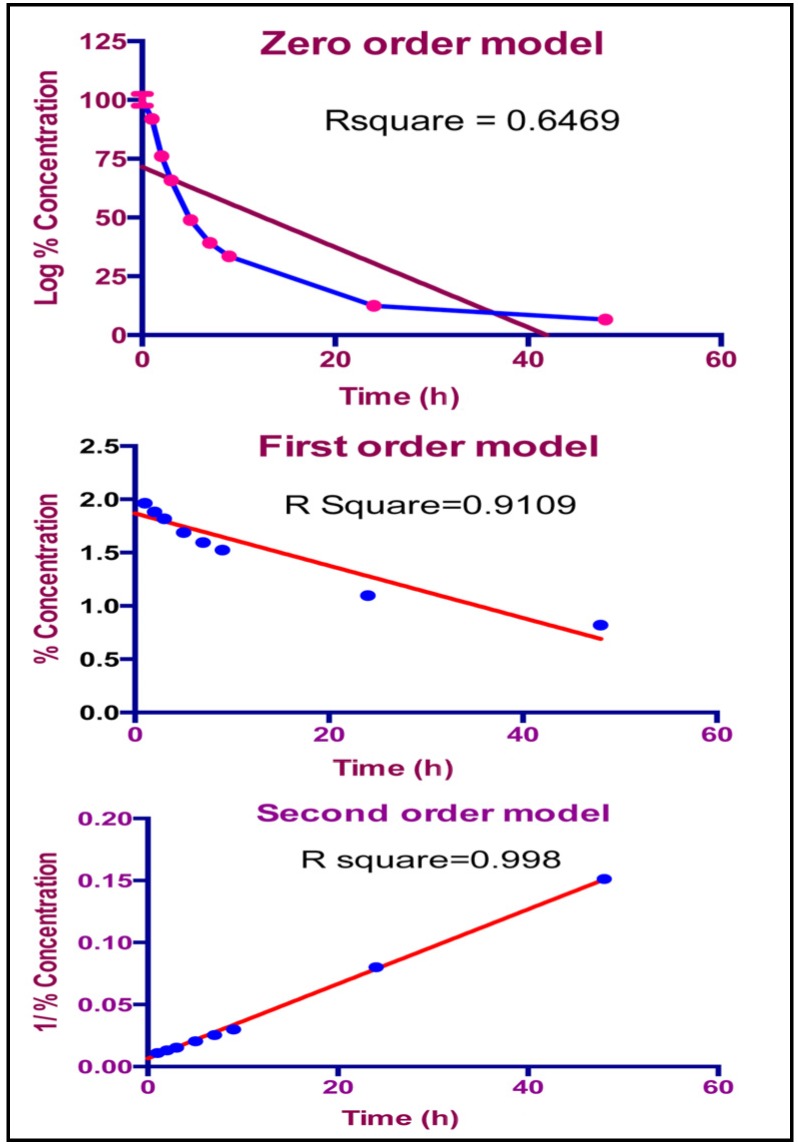
TQ Light degradation kinetics plotted for zero, first and second order models.

In this study, the effect of light on the integrity of TQ was investigated and the results clearly show that TQ was highly sensitive to light, even after a short period of exposure. Light sensitivity of TQ was found to be independent of the solvent type. In general, for all solvents, the results indicate that less than 20% of the drug remained intact after 24 h and this decreased further to less than 10% after 48 h. To study the kinetics of the photolysis, TQ in ethanol was monitored over shorter time periods. Results in Figure 6 depict rapid degradation of TQ (more than 70%) within the first 10 h, and the process was also observed to follow second order kinetics (R^2^ = 0.998).

**Figure 7:**
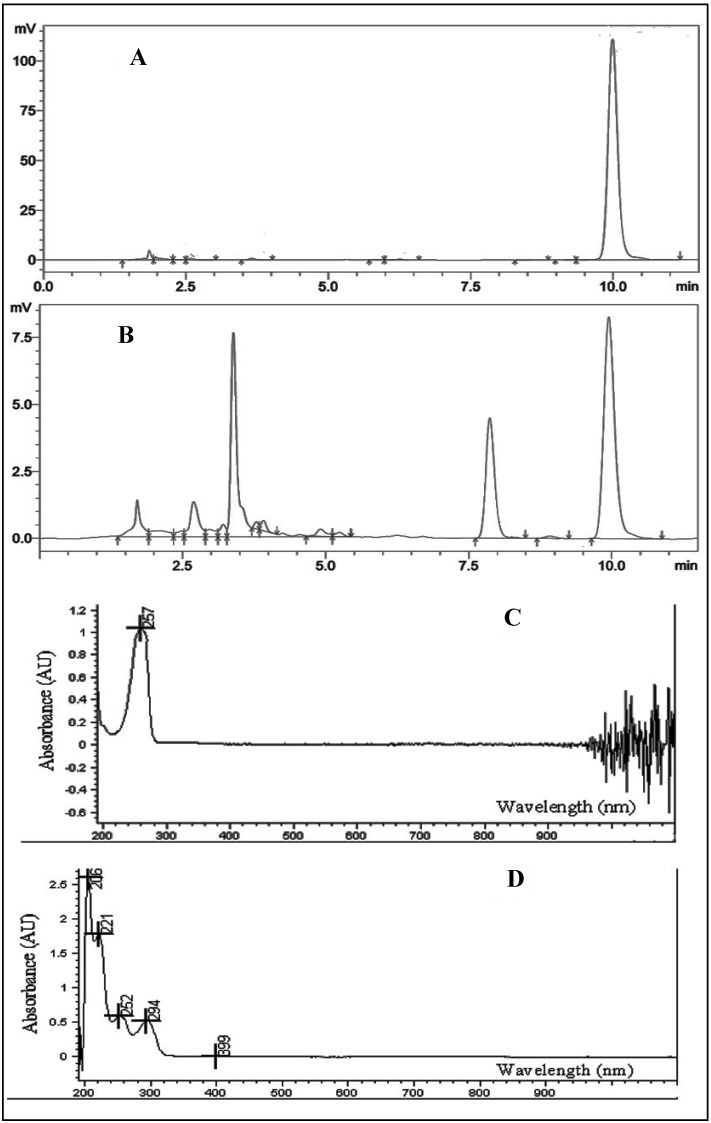
Initial HPLC chromatographs of TQ (**A**), after 48 h light stability study (**B**) and the initial UV-VIS spectrum of TQ (**C**), after 48 h light stability study (**D**).

Four main light degraded products were observed after 48 h in the HPLC chromatograms (Figure 7A,B). All products were well resolved from TQ and can be safely assumed to be more hydrophobic due to their shorter retention times.

UV-VIS absorption spectroscopy is one of the best methods for the determination of impurities in organic molecules. Additional peaks can be observed due to impurities in the sample and it can be compared with that of standard raw material. The initial UV-VIS analysis of TQ compared to that after 48 h (Figure 7C,D) showed very interesting changes; in addition to the disappearance of the TQ peak at 257 nm, a few more peaks with different λ_max_ appeared in the spectrum. These observations confirm the degradation process and the formation of new compounds.

It has been reported by Orlando *et al.* that prior to their research, the photochemical reactions of TQ had been primarily concerned with dimerization, but they showed that the isopropyl side chain in TQ undergoes the same type of photorearrangement as the *t*-butyl group in *t*-butyl-*p*-benzoquinone. Probably a spirocyclopropyl intermediate is involved in both cases. They were able to separate at least three main new compounds with good yield [22].

On the other hand, samples stored under dark conditions retained the initial concentration of TQ even after 48 h. Furthermore, stability in absence of light was also monitored for 1:1 combinations of ethanol with water or phosphate buffer. There were no considerable changes in TQ concentration even after 96 h, indicating higher stability of TQ in these solvents. These results suggest that combination of organic and aqueous solvent in a 1:1 ratio may appear as a good alternative to pure aqueous media for the testing the *in vitro* drug release in case of compatibility with the formulation being tested. On the basis of these findings, we strongly recommend protection of all TQ solutions from light during the formulation process to ensure a stable drug for quantification, which in turn may help in exploring the full potential of this drug.

## 3. Experimental

### 3.1. Qualitative Analysis of Thymoquinone

Thymoquinone (99% purity) was purchased from Sigma Aldrich (St. Louis, MO, USA). UV-VIS spectrophotometry is routinely used in analytical chemistry for the qualitative determination of different analytes. The UV-VIS spectrophotometric analyses of TQ were performed on a Agilent-8453 (Santa Clara, CA, USA) UV-VIS spectrophotometer.

### 3.2. Quantitative Analysis of Thymoquinone

The quantitative analysis was conducted on HPLC system (Shimadzu LC-2010C, Kyoto, Japan) equipped with an autosampler, and a diode array detector. Chromatographic separation was performed on a Hedera ODS-2 column (4.6 × 250 mm, 5 μm). TQ was detected using the method described by El-Najjar N. *et al.* [23], with minor modifications. The samples were eluted using an isocratic mobile phase of water/ACN (40:60% *v*/*v*) at a flow rate of 1 mL/min. The diode array detector signal was recorded at 257 nm, and the injection volume was 50 μL. The chromatographic data was acquired and analyzed using the Shimadzu LC solution software package. The HPLC method was subjected to validation as per the International Conference on Harmonization (ICH) regulations Q2 (R1) 2005 to make sure of the validity of the method despite the minor changes we made. The Limit Of Detection (LOD) and Limit Of Quantitation (LOQ) calculated from the calibration curve as LOD = 3.3 σ/S, LOQ = 10 σ/S where σ is the standard deviation of the y-intercepts and S is the slope of the calibration curve.

According to the ICH Guidance for Industry Q1 A (R2) Stability Testing of New Drug Substances and Product Examination, the degradation products under stress conditions are useful in establishing degradation pathways and developing and validating suitable analytical procedures.

A forced degradation study as previously reported [21] was performed for TQ and it was subjected to HPLC analysis to confirm the method ability for detecting the drug degradation products and to be qualified as a stability indicating method for TQ detection and quantification in pharmaceutical preparations.

### 3.3. Solubility Study

The study was carried out adding excess amounts of TQ in 10 mL of a series of aqueous solutions with different pH values in well closed amber containers that were agitated at 37 °C for 24, 48, and 72 h. The samples were then filtered through a 0.22 μm membrane filter, and the drug content was measured by HPLC after appropriate dilution. All experiments were done in triplicate.

### 3.4. Aqueous Stability Study

The degradation study was performed in 0.1 N HCl aqueous solution, water, saline and buffer solutions (pH 5, 7.4, and 9) with concentrations of 50 mM at 37 °C. Stock solution of TQ (1 mg/mL) in ethanol was diluted with the specified media to give a final concentration of about 60 μg/mL and the solutions were kept in a shaking water bath in tightly closed amber containers. At appropriate time intervals, aliquots were withdrawn, filtered and properly diluted with mobile phase and injected into the HPLC system for the determination of TQ content. The rate constants were determined from the slopes of the best-fit linear plots model of the remaining drug *versus* time. The HPLC chromatograms were compared to evaluate the extent of degradation. Experiments were triplicated and the values were expressed as mean ± SD.

### 3.5. Effect of Light and Solvent Type on the Degradation of TQ

To study the degradation kinetics due to light in different solvents, fixed amounts of TQ stock solution in ethanol (1 mg/mL) were diluted with three different solutions (ethanol, ethanol/water or pH 5 buffer 1:1) to give a final concentration of 100 μg/mL. Then the solutions were divided into two sets. One set exposed to light in an illumination chamber with an illuminance of 750 lux at 25 °C, another set was kept under dark at the same temperature. Samples were withdrawn, filtered and properly diluted and checked for drug content using HPLC throughout a period of 48 h, also the UV-VIS spectra was recorded before and after the study to monitor the possible changes. The kinetics of the degradation rate was then calculated from the best-fit model. The experiments were run in triplicate and values were expressed as mean ± SD.

## 4. Conclusions

In this research we studied the solubility and stability of TQ in different aqueous solutions and the study revealed that the drug might already have enough aqueous solubility to induce *in vivo* therapeutic effects, particularly if administered via a parenteral route. Unfortunately, the solubility of the drug is compromised by its stability problems, as we also demonstrated that TQ is highly unstable in aqueous solutions with prominent effects of both pH and light, where the latter has the greater effect. In addition the discrepancies in previously published reports about TQ that have been pointed out and correlated to stability problems, this data confirmed the unsuitability of pure aqueous solutions as pharmaceutical vehicles of TQ preparations; instead the combination of organic and aqueous solvents seemed to be good alternative. The current research may contribute to enrich the knowledge of the physicochemical properties of this drug for exploring its full anticancer potential in the future. It would also facilitate the process of formulation of TQ in durable stable preparations that may fill the gap between the already confirmed *in vitro* efficacy and the anticipated clinical use of this valuable anticancer drug.
